# Food label literacy among Malaysian school adolescents: A prevalence study

**DOI:** 10.1371/journal.pone.0324142

**Published:** 2025-05-29

**Authors:** Syaza Kamarudin, Bee Koon Poh, Hanis Mastura Yahya, Norazmir Md Nor, Raduan Sharif, Ruzita Abd Talib

**Affiliations:** 1 Center for Community Health Studies (ReaCH), Faculty of Health Sciences, Universiti Kebangsaan Malaysia, Kuala Lumpur, Malaysia; 2 Nutritional Sciences Programme, Faculty of Health Science, Universiti Kebangsaan Malaysia, Kuala Lumpur, Malaysia; 3 Center for Healthy Ageing and Wellness (H-CARE), Faculty of Health Sciences, Universiti Kebangsaan Malaysia, Kuala Lumpur, Malaysia; 4 Centre for Nutrition and Dietetics Studies, Faculty of Health Sciences, Universiti Teknologi MARA, Selangor, Malaysia; 5 Mass Communication, Faculty of Creative Industries, Universiti Tunku Abdul Rahman, Selangor, Malaysia; Universiti Putra Malaysia, MALAYSIA

## Abstract

Making informed food choices is crucial for all ages, especially adolescents who are involved in selecting and buying food. Understanding nutrition labels helps guide choices and ensures nutritional needs are met. This study aims to assess the prevalence and level of food label literacy among school-going adolescents aged 13–16 in Malaysia. This study involved a cross-sectional online survey and was sampled by random sampling involving secondary school adolescents aged 13–16, totaling 1983 subjects across five regions in Malaysia: Central, Southern, Northern, and East Coast of Peninsular Malaysia, as well as East Malaysia (Sabah and Sarawak). The questionnaire includes sections on sociodemographic and anthropometric information, as well as three main food label literacy domains: comprehension and interpretation of food labels, skills in using food labels, and attitudes towards food labels. A total of 89.7% of adolescents read food labels with a moderate level of literacy across all three domains (comprehension and interpretation, skills in usage, and attitudes) classified using predefined score ranges based on previous studies. A significant relationship (p < 0.05) was found between age and body mass index-for-age z-score (BAZ) with the level of food label literacy, indicating higher literacy values in older adolescents. Mean scores of all domains A, B, and C across the five regions indicated significant differences at F(4,1978) = 25.8, p = 0.000, F(4,1978) = 17.43, p = 0.000, and F(4,1978) = 25.20, p = 0.000. In conclusion, the proportion of Malaysian adolescents reading food labels is high (89.7%). Nevertheless, the food label literacy level across all three domains is moderate. Hence, the promotion of the importance of nutrition labels in schools through organised programs such as interactive hands-on label reading activities and school-based nutrition campaigns are recommended. A crucial gap in health education can be addressed by fostering proper comprehension, skills, and attitudes toward food labels and facilitating informed dietary practices among adolescents.

## Introduction

Adolescence is the transitional phase between childhood and adulthood and encompasses significant cognitive, psychological, and physical changes [1]. This pivotal period encourages adolescents to cultivate resilience, assertiveness, and social interaction skills, which are essential for navigating the complexities of the world around them [1]. Central to their development is the critical need for nutritious food to ensure healthy growth and well-being [2]. However, in today’s digital era, social media trends exert substantial influence over dietary choices [3], often overshadowing nutritional considerations [4].

The proliferation of viral content and trending topics on social media platforms has reshaped societal norms, including food preferences and consumption habits [5]. Viral videos, such as product introductions for new food items, captivate audiences and can influence widespread dietary behaviours [6]. This remains relevant, as a recent study states that food and beverage marketing significantly influence the development of a preference for and desire to consume energy-dense, low-nutrient foods [7]. Recent Malaysian studies highlight social media’s impact on food choices among youth. Kei (2024) [8] found that Instagram influencers play a key role in promoting healthier eating. Additionally, Salleh et al. (2021) [9] explored how social media impacts food popularity among Malaysian students, finding that YouTube had the greatest influence due to its visual appeal, which significantly shaped students’ food preferences. These studies underscore social media’s growing influence on Malaysian adolescents’ food choices. Consequently, the focus on nutritional content in food selection has diminished despite the presence of food labels intended to guide informed choices [10].

Food label literacy refers to an individual’s capacity to obtain, comprehend, and utilise information found on nutrition labels [11]. It emerges as a critical skill in the midst of evolving dietary landscapes [12]. Asakura et al. (2017) [13] emphasised that high nutritional knowledge, a foundation of food label literacy, is linked to healthier eating patterns among children. However, the increasing prevalence of obesity and related non-communicable diseases among them highlights the urgency of promoting health literacy, particularly concerning dietary practices [14]. Those who interpret food labels accurately can incorporate healthy eating habits into their daily lives [15].

In Malaysia, 84% of adolescents engage with product labels, yet only 33% routinely reading labels, focusing primarily focusing on the expiration dates [16]. As agents of change, adolescents play a pivotal role in shaping the future of society and the nation [17]. This phase of life is not only a period of personal growth but also a crucial time for societal development [17], making it essential to equip them with strong health literacy skills. according to the WHO’s holistic definition of health which encompasses physical, mental, and social well-being, health literacy is a fundamental component of overall well-being [14]. This aligns with the growing body of literature on nutrition and food literacy, which underscores the importance of developing health literacy skills in relation to food [18]. As outlined by Miller (2016) [19], food label literacy is an integral part of health literacy.

In light of this, one proactive step towards making informed food choices and maintaining proper nutrition is to read the nutritional labels on the food products one intends to purchase. By fostering comprehension, skills, and attitudes toward food labels, a crucial gap in health education can be addressed, facilitating informed dietary practices among adolescents, as stated by Janeta and Santoso (2018) [20], i.e., the quality of life can be determined by an individual’s food choices.

Given the significance of this issue, the present study aims to assess food label reading and literacy among adolescents aged 13–16 years in Malaysia. Furthermore, this study seeks to identify the influence of sociodemographic factors on food label literacy, building upon more information to highlight the significant associations. Nowadays, understanding and promoting food label literacy are proactive steps to empower adolescents to make informed and healthier food choices, thereby contributing to their overall well-being and societal advancement.

## Methods

### Design, location, and time

This study incorporated an online cross-sectional study involving Malaysian adolescents between the ages of 13 and 16 years who attended schools across five regions of Malaysia (Central, East Coast, Southern, Northern, and East Malaysia). Recruitment and data collection were conducted from 25th September 2023–31st December 2023. This study was approved by The Research Ethics Committee, The National University of Malaysia (Reference No: JEP-2023–544). Online informed consent was obtained from the participants’ parents or legal guardians via Google Forms prior to their participation in the study. Although the study period was relatively short (September to December 2023), the data collection process was carefully structured to ensure a representative sample across the regions, school settings, and demographic groups. Since food label literacy is not influenced by seasonal variations, the findings are considered generalizable within the study population.

### Sampling and tools

Sample selection utilised multi-stage cluster sampling across three primary clusters: regions, district education offices (PPDs), and schools. A simple random sampling, employing a lottery method, was then conducted within each selection. Malaysia was divided into five geographical zones, with two states per zone randomly selected via a lottery method, resulting in ten states. Next, one PPD per selected state was randomly chosen, totaling 10 PPDs. Within each PPD, schools were proportionally sampled based on student registration numbers. To ensure an unbiased selection, a computerized random selection process was used at each stage, maintaining the integrity of random sampling while accommodating the online recruitment process. Finally, students were randomly selected within schools using a lottery method, following inclusion and exclusion criteria. Since the number of schools and students varied by state, their selection was proportionally allocated based on the total sample size calculated. Both urban and rural areas were included in the sampling process to ensure diverse representation. Participants provided online informed consent, which was filled out by their parents through Google Forms, before commencing the study. The questionnaire was then distributed to the participants through the same online platform. Participants who were able to read and write were considered eligible for inclusion in this study. Meanwhile, the exclusion criteria encompassed individuals with physical disabilities that could potentially disrupt accurate weight and height measurements.

The questionnaire utilised in this study was structured based on the research by Ramdan et al. (2018), who investigated the relationship between food label literacy, attitudes toward nutrition labels, and the selection of healthy foods. Additionally, questions on food label literacy and the ability to read food label panels were derived from the study by Jefrydin et al. (2019) [21]. The constructed questionnaire, comprising four sections, was validated for use among Malaysian adolescents to assess food label literacy among participants in this study. Prior to the study, the questionnaire, was validated for use among Malaysian adolescents to assess food label literacy. Face and content validity were assessed by a panel of academic nutrition experts and school educators to ensure clarity, relevance, and suitability. To assess the reliability, a pilot study was conducted among a subset of adolescents. The questionnaire reliability was then evaluated using Cronbach’s alpha test. The values were 0.952, 0.879 and 0.953 for food label comprehension and interpreting skills, skills in using food labels and attitudes towards food labels respectively. According to Ahmad et al. (2024) [22], achieving a Cronbach’s alpha of at least 0.70 is indicative of reliable internal consistency, making it suitable for use in research studies.

Section A comprises sociodemographic information and food labelling usage practice, while the remaining sections assess food label literacy across three domains. The first domain, Section B evaluates food label comprehension and interpreting skills, measuring participants’ ability to understand the information on nutrition labels and apply it to food choices. The second domain, covered in Section C, examines skills in using food labels, assessing how effectively participants utilize label information in decision-making. Lastly, the third domain, covered in Section D, assesses attitudes towards food labels, measuring the subjects’ capability to utilise the information on nutrition labels to make informed decisions about their food choices. Food label comprehension and interpreting skills (Section B), and attitudes towards food labels (Section D) were classified using a min-range approach as suggested by Benitez (2022)ranging from 1.00–2.71 indicate low literacy, 2.72–5.29 indicate moderate literacy, and 5.30–7.00 indicate high literacy [23], while skills in using food labels (Section C) were assessed using percentage scores (Low: 0–49%, Moderate: 50–79%, High: 80–100%) [24].

### Data collection

The data for this study were collected through an online questionnaire created using Google Forms, which contained information on sociodemographic characteristics (gender, age, parental employment status, parental educational attainment, parental marital status, and household income). Additionally, data on anthropometric measurements (weight and height) were derived from the National Physical Fitness Standards for Malaysian School Students (SEGAK) records and the participants’ body mass index-for-age z-score (BAZ) was computed using the WHO AntroPlus software, with cut-off points according to the z-score of -3 SD (severe thinness), -2 SD (thinness), -1 SD until +1 SD (normal), + 1 SD (overweight), and +2 SD (obese). The sample size was determined using the single proportion formula to estimate prevalence [25] based on the enrolment data of Malaysian secondary school students sourced from the [26]. A total of 2,039 subjects were required for this survey, accounting for a 50% dropout rate, representing the nationwide student population. However, only 1983 subjects completed the survey.

### Data analysis

The data were analysed using IBM SPSS Statistics for Windows version 25.0 (IBM, Armonk, New York, USA). Parametric tests, including one-way ANOVA and independent t-tests, were employed for analysis. Descriptive statistics were utilised to determine the sociodemographic data and food label reading status, providing both numerical counts and percentages. Food label literacy levels were classified using a min-range approach for comprehension and interpreting skills, as well as attitudes, while skills in using food labels were assessed using percentage scores.

## Results

Table 1 illustrates the sociodemographic characteristics of the study participants. A total of 1,983 subjects were included, comprising four age groups: 13 years old (406 subjects), 14 years old (452 subjects), 15 years old (542 subjects), and the majority at 16 years old (583 subjects). On average, the subjects are 14.7 ± 1.1 years old. The sample included 662 males and 1321 females. The study participants were drawn from five primary regions across Malaysia: Central, Southern, Northern, and East Coast of Peninsular Malaysia, and East Malaysia (Sabah and Sarawak), with participation percentages of 28.1%, 22.2%, 15.6%, 15.7%, and 18.4%, respectively. Approximately 77.9% of the participants resided in urban areas, while 22.1% were from rural areas. The ethnicity of the subjects is categorised into five groups: Malay, Chinese, Indian, Sabah Bumiputera, and Sarawak Bumiputera. The largest ethnic group among the subjects is Malay (59.3%), followed by Chinese (16.8%), natives of Sabah (12.1%), natives of Sarawak (6.4%), and Indian (5.3%). This is attributed to the fact that although the Malaysian population comprises three major ethnic groups‒the Bumiputera (i.e., the Malays and indigenous people), Chinese, and Indian, the Malay ethnic group is among the majority at 69.4%, followed by the Chinese at 23.2%, and the Indian at 6.7% [27].

**Table 1:** Subject’s sociodemographic characteristics.

Sociodemographic factors	N	%	Mean ± SD
**Age**			14.7 ± 1.1
13 years	406	20.5	
14 years	452	22.8	
15 years	542	27.3	
16 years	583	29.4	
**Gender**			
Boys	662	33.4	
Girls	1321	66.6	
**Ethnicity**			
Malay	1176	59.3	
Chinese	334	16.8	
Indians	106	5.3	
Bumiputra Sabah	240	12.1	
Bumiputra Sarawak	127	6.4	
**Socioeconomic Status**			4531.0 ± 8292.3
B40 (<RM5250)	1455	73.4	
M40 (RM5250– RM11819)	399	20.1	
T20 (>RM11819)	129	6.5	
**Parent’s Marital Status**			
Married	1728	87.1	
Divorced/Widowed	255	12.9	
**Mother’s Education Level**			
Institutes of higher education	669	33.7	
Primary/secondary school	1267	63.9	
Does not go to school	47	2.4	
**Father’s Education Level**			
Institutes of higher education	596	30.1	
Primary/secondary school	1340	67.6	
Does not go to school	47	2.4	
**Region**			
Central	557	28.1	
Southern	312	15.7	
Northern	364	18.4	
East Coast	310	15.6	
East Malaysia	440	22.2	
**Area of Residence**			
Urban	1545	77.9	
Rural	438	22.1	

The classification for socioeconomic status is based on the Department of Statistics Malaysia (DOSM).

RM: Ringgit Malaysia. 1 US dollar = 4.04 MYR (as of January 30, 2021)

In terms of socioeconomic status, over 73.4% of all subjects are categorised under the B40 group. The B40 cluster represents the bottom 40% of income earners, comprising individuals with household incomes below RM5,250 per month. Subsequently, 20.1% of the subjects belong to the M40 group, i.e., the middle 40%, which includes household incomes ranging from RM5,250 to RM11,819. Meanwhile, only 6.5% fall into the T20 group, i.e., the top 20%, comprising individuals with household incomes exceeding RM11,820 per month. According to the Household Income Survey 2022 [27], the T20 group constitutes the majority of household income categories in Malaysia, at 46.3%. In this study, the mean for household income is 4531.0 ± 8292.3. Most of the participants’ parents’ marital status is predominantly married, with 1,728 individuals, while 255 individuals are either divorced or widowed. Regarding the educational attainment of fathers, 67.6% have completed primary or secondary education, 30.1% have completed higher education, and only 2.4% have no educational background. Similarly, for mothers, 63.9% have completed primary or secondary education, 33.7% have completed higher education, and 2.4% have no education educational background.

Table 2 illustrates the BAZ distribution of the subjects. The majority of the subjects are in the normal BAZ category (65.9%), followed by those categorised as overweight (17.2%), obese (9.7%), and thin (5.3%). The smallest proportion of subjects fell into the severe thinness category, comprising only 2.0%. On average, the subjects’ BAZ is 21.0 ± 4.82 kg/m².

**Table 2:** Subject’s body-mass-index-for-age z-score categories.

Body Mass Index (BMI)	N	%	Mean ± SD
Severe Thinness	39	2.0	21.0 ± 4.82
Thinness	105	5.3	
Normal	1306	65.9	
Overweight	341	17.2	
Obese	192	9.7	

The findings in Table 3 reveal that 89.7% of Malaysian adolescents read food labels, while 10.3% reported never reading them. Additionally, the data indicate that a slightly higher percentage of girls (90.3%) read food labels compared to boys (88.4%). Conversely, 9.7% of girl subjects and 11.6% of boy subjects reported never reading food labels.

**Table 3:** Food label reading habits of Malaysian adolescents.

	Overall	Male	Female
	% (N)	% (N)	% (N)
**Food Label Reading Habits**			
Yes	89.7 (1778)	88.4 (585)	90.3 (1193)
No	10.3 (205)	11.6 (77)	9.7 (128)

Table 4 illustrates the food label literacy level among Malaysian school adolescents, evaluated based on food label literacy domains. The primary outcome indicates that, overall, Malaysian adolescents demonstrate a moderate level of proficiency across all assessed domains: understanding and interpreting food labels, skills using food labels, and attitude toward food labels, with the means for each domain of 4.66 ± 1.24, 65.8 ± 21.3, and 4.97 ± 1.23, respectively. However, upon comparison among the five regions, while the results are consistently moderate, the central region exhibits the lowest means across all domains: understanding and interpreting food labels (4.35 ± 1.43), utilising food labels (59.7 ± 20.1), and attitude toward food labels (4.63 ± 1.18) compared to other regions. Additionally, the East Coast region is noted with the highest mean in understanding and interpreting food labels, whereas the Northern region demonstrates the highest mean in skills in using food labels as well as an attitude toward food labels, with means of 69.3 ± 19.9 and 5.26 ± 1.25. Other than that, the results indicate significant differences in all domains: Domain A – F(4,1978) = 25.8, p < 0.0001, Domain B – F(4,1978) =17.43, p < 0.001, and Domain C – F(4,1978) = 25.20, p < 0.001.

**Table 4:** Food label literacy level of Malaysian adolescents by region.

Region	Domain A Understanding & Interpreting Food Labels			Domain B Skills Using Food Labels			Domain C Attitudes Toward Food Labels		
	Mean ± SD			Mean ± SD			Mean ± SD		
Overall	4.66 ± 1.24 Moderate			65.8 ± 21.3 Moderate			4.97 ± 1.23 Moderate		
	Mean ± SD	F	p	Mean ± SD	F	p	Mean ± SD	F	p
Central	4.35 ± 1.43 Moderate	25.80	.000*	59.7 ± 20.1 Moderate	17.43	.000*	4.63 ± 1.18 Moderate	25.20	.000*
Southern	4.78 ± 1.04 Moderate			66.3 ± 24.8 Moderate			4.76 ± 1.13 Moderate		
Northern	4.86 ± 0.73 Moderate			69.3 ± 19.9 Moderate			5.26 ± 1.25 Moderate		
East	5.10 ± 1.13 Moderate			66.86 ± 21.8 Moderate			5.12 ± 1.29 Moderate		
East Malaysia	4.45 ± 1.39 Moderate			69.3 ± 19.3 Moderate			5.22 ± 1.19 Moderate		

The p-value was obtained using a one-way ANOVA test. Significant at p < 0.05

The p* value indicates significance

Table 5 depicts the comparison between all three domains: understanding and interpretation of nutrition labels, skills in using nutrition labels, and attitudes toward food labels among various sociodemographic factors, including age, gender, parental marital status, parental education, family economic status, and BAZ. Upon analysis of the p-value results for domains A and B (understanding and interpreting food labels and skills using food labels), all sociodemographic factors exhibit non-significant values (p > 0.05), except for age and BAZ. For age, the results show a p-value of p < 0.001 for both understanding and interpretation of nutrition labels, as well as skills in using nutrition labels. Consequently, age emerges as the sole significant factor influencing both the understanding and interpretation, as well as skills in using nutrition labels among the specified sociodemographic factors. In the comparison of attitudes toward food labels with various sociodemographic factors, a notable p-value (p < 0.05) is noted only in the comparison based on the father’s education level. This suggests that a father’s education level may play a significant role in shaping adolescents’ attitudes toward food labels, highlighting the potential influence of the father’s educational background on nutritional literacy and decision-making behaviours among adolescents. When comparing food label literacy across socioeconomic groups, a significant difference was found in the understanding and interpretation of food labels across socioeconomic groups (p < 0.05), with adolescents from higher-income households demonstrating better comprehension. However, there were no significant differences in skills using food labels or attitudes toward them (p > 0.05

**Table 5:** Comparison between food label literacy domains with sociodemographic factors.

Domains	Understanding & Interpreting Food Labels			Skills Using Food Labels			Attitudes Toward Food Labels		
Sociodemographic factors	Mean ± SD	F	p	Mean ± SD	F	p	Mean ± SD	F	p
**Age**		7.757	.000^***^		17.684	.000^***^		3.480	.015^*^
13 years	4.45 ± 1.32			59.54 ± 21.88			4.85 ± 1.32		
14 years	4.63 ± 1.29			65.57 ± 20.54			4.91 ± 1.19		
15 years	4.65 ± 1.23			66.79 ± 20.27			5.08 ± 1.17		
16 years	4.83 ± 1.15			69.28 ± 21.52			5.01 ± 1.25		
**Sex**		4.463	.035^*^		1.796	.180		3.062	.080
Boys	4.57 ± 1.29			65.28 ± 22.22			4.97 ± 1.29		
Girls	4.70 ± 1.22			66.00 ± 20.83			4.98 ± 1.20		
**Parents’ Marital Status**		3.192	0.74		.130	.718		.049	.825
Married	4.62 ± 1.26			65.68 ± 21.33			4.96 ± 1.24		
Divorced/Widowed	4.91 ± 1.14			66.28 ± 21.16			5.05 ± 1.20		
**Father’s Education Level**		1.062	.346		2.397	.091		5.666	.004^**^
Higher education	4.64 ± 1.33			66.36 ± 21.52			4.83 ± 1.21		
Primary/secondary school	4.66 ± 1.21			65.72 ± 21.24			5.04 ± 1.24		
Does not go to school	4.91 ± 1.18			59.31 ± 19.59			4.98 ± 1.23		
**Mother’s Education Level**		.804	.448		2.602	.074		4.469	.012^*^
Higher education	4.65 ± 1.29			66.03 ± 21.98			4.86 ± 1.25		
Primary/secondary school	4.65 ± 1.23			65.87 ± 20.97			5.03 ± 1.22		
Does not go to school	4.88 ± 1.11			58.78 ± 19.49			4.98 ± 1.23		
**Socioeconomic Status**		3.919	.020^*^		.382	.683		2.940	.053
B40 (<RM5250/USD1119)	4.67 ± 1.21			65.52 ± 21.12			5.01 ± 1.24		
M40 (RM5250/USD1119 – RM11819/USD 2,519)	4.53 ± 1.33			66.57 ± 21.43			4.84 ± 1.17		
T20 (>RM11819/USD2519)	4.86 ± 1.34			65.89 ± 23.00			5.00 ± 1.32		
**Body Mass Index**		2.652	.032^*^		3.549	.007^*^		1.181	.230
Severe Thinness	4.80 ± 1.04			47.59 ± 18.90			4.87 ± 1.12		
Thinness	4.72 ± 1.19			47.79 ± 21.16			4.87 ± 1.32		
Normal	4.69 ± 1.25			41.46 ± 24.84			5.01 ± 1.22		
Overweight	4.46 ± 1.26			38.58 ± 24.63			4.85 ± 1.28		
Obese	4.68 ± 1.23			41.10 ± 24.14			5.03 ± 1.20		

The p-value was obtained using a one-way ANOVA test and an independent T-test. Significant at p < 0.05

The p value with an asterisk * indicates significance

*p < 0.05

**p < 0.01

***p < 0.001

RM: Ringgit Malaysia. 1 US dollar = 4.04 MYR (as of January 30, 2021)

Table 6 presents the results of the Games-Howell post hoc test, which was conducted to identify pairwise differences in Comprehension & Interpreting Food Labels, Skills Using Food Labels, and Attitudes Toward Food Labels across different regions. Only significant comparisons (p < 0.05) are reported. For Comprehension & Interpreting Food Labels, significant regional differences were observed. Central scored significantly lower than Southern (M = -0.4391, p = 0.000), Northern (M = -0.5194, p = 0.000), and East (M = -0.7580, p = 0.000). Additionally, Southern had significantly lower scores than East (M = -0.3188, p = 0.003) and East Malaysia (M = -0.3159, p = 0.004). Lastly, East scored significantly lower than Northern (M = 0.2385, p = 0.014). For Skills Using Food Labels, Central demonstrated significantly lower scores compared to Southern (M = -5.4671, p = 0.008), East (M = -9.5822, p = 0.000), and East Malaysia (M = -7.1376, p = 0.000). Similarly, Northern outperformed Central (M = -9.5441, p = 0.000), while East (M = 9.5822, p = 0.000) and East Malaysia (M = 7.1376, p = 0.000) also demonstrated significantly higher scores. For Attitudes Toward Food Labels, Central had significantly lower scores than Northern (M = -9.5441, p = 0.000), East (M = -0.6437, p = 0.000), and East Malaysia (M = -0.4942, p = 0.000). Similarly, Southern scored significantly lower than Northern (M = -0.5949, p = 0.000) and East (M = -0.5086, p = 0.000). Additionally, East Malaysia exhibited significantly lower scores compared to Central (M = -0.3591, p = 0.003).

**Table 6:** Post-hoc comparison of food label literacy domains across zones.

Dependent Variable	Comparison (I-J)	Mean Difference (I-J)	Std. Error	p-value	95% Confidence Interval
Comprehension & Interpreting Food Labels	Central - Southern	-0.4391^*^	0.08447	0.000	-0.6701, -0.2083
	Central - Northern	-0.5194^*^	0.07142	0.000	-0.7147, -0.3242
	Central - East	-0.7580^*^	0.08885	0.000	-1.0010, -0.5151
	Southern – East	-0.3188^*^	0.08786	0.003	-0.5592, -0.0785
	Southern – East Malaysia	-0.3159^*^	0.08884	0.004	-0.0731, -0.5589
	East - Northen	0.2385^*^	0.07541	0.014	0.0322, 0.4450
Skills Using Food Labels	Central - Southern	-5.4671^*^	1.6415	0.008	-9.960, -0.974
	Central - East	-9.5822^*^	1.3460	0.000	-13.262, -5.902
	Central - East Malaysia	-7.1376^*^	1.5010	0.000	-11.245, -3.031
	Northern – Central	-9.5441^*^	1.2528	0.000	-12.698, -6.390
	East – Central	9.5822^*^	1.3460	0.000	5.902, 13.262
	East Malaysia - Central	7.1376^*^	1.5010	0.000	3.031, 11.245
Attitudes Toward Food Labels	Central - North	9.5441^*^	1.2528	0.000	6.120, 12.968
	Central - East	-0.6437^*^	0.08225	0.000	-0.8686, -0.4188
	Central - East Malaysia	-0.4942^*^	0.08889	0.000	-0.7375, -0.2511
	Southern - Northern	-0.5949^*^	0.07543	0.000	-0.8011, -0.3888
	Southern - East	-0.5086^*^	0.09148	0.000	-0.7588, -0.2583
	East Malaysia - Central	-0.3591^*^	0.09750	0.003	-0.6259, -0.0924

Only significant comparisons are shown (p < 0.05).

*p < 0.05

## Discussion

The percentage of adolescents reading nutrition labels in this study is slightly higher at 89.7% compared to 84% reported in NHMS 2017 [16]. This may be due to effective educational programs, changes in dietary trends, or increased access and awareness of nutrition labels [27–29]. However, when comparing the actual number of adolescents who always read food labels, this study showed a slightly lower percentage of 23.5% compared to 33% in NHMS 2017. Additionally, 66.2% reported sometimes reading food labels in this study, whereas only 51% reported sometimes reading food labels in NHMS 2017.

In relation to the decreasing percentage of subjects who always read food labels, Jefrydin et al. (2019) [21] identified several barriers that hinder adolescents from doing so. Among them is the lack of interest in reading food labels, considering food labels unimportant, especially for familiar foods, and cravings for certain foods, which led them to ignore labels in favour of immediate satisfaction. Other than that, many adolescents find food labels complex and difficult to understand, which discourages their regular use [30]. Moreover, time constraints further deter adolescents from reading food labels thoroughly, and they tend to prioritise food based on packaging rather than nutritional value [31]. These factors were also discussed in NHMS 2017 and may be the primary reasons for the decrease in the number of adolescents who always read food labels.

A one-way ANOVA test reveals that the mean scores across all three domains for all five regions in Malaysia, Central, Southern, Northern, East Coast, and East Malaysia, are at a moderate level. This finding contradicts the study by Jefrydin et al. (2019) [21], who reported that food label utilisation was low. The East zone has the highest mean score for domain A, i.e., understanding and interpretation of food labels (5.10 ± 1.13), indicating that adolescents in this zone have a better understanding and interpretation of food labels than other zones. However, the Northern zone scored the highest in domain B, i.e., skills in using food labels (18.9 ± 3.7) and domain C, i.e., attitudes towards food labels (5.26 ± 1.25). The results signify that adolescents in these zones are more proficient in using nutrition labels and can utilise the information on nutrition labels to make informed food choices.

While Malaysian adolescents demonstrate a moderate level of food label literacy, comparisons with other populations reveal varying levels of nutrition literacy among adolescents globally. For instance, a large-scale study across ten Arab nations found that 28% of adolescents had poor nutrition literacy, with the highest prevalence observed in Qatar (44%), Lebanon (37.4%), and Saudi Arabia (34.9%) [32]. Similarly, research in Kuwait reported that while 72.5% of adolescents had median overall nutrition literacy, 73.3% exhibited inadequate understanding of food labels [33]. In Turkey, moderate nutrition literacy levels were observed, with dietary habits and food label use playing a significant role in influencing literacy [34]. In Korea, food literacy was explored in relation to adolescent food-related lifestyles, media use, and eating behaviors, further emphasizing the role of nutrition education in dietary habits and the finding reveals that the average food literacy score was 55.57 points which is moderate [35]. These comparisons highlight the need for targeted interventions to enhance food label literacy among adolescents globally, considering cultural and regional dietary influences. Studies in other Asian countries, such as Thailand and Indonesia, have shown the positive impact of nutrition education on adolescents’ food label literacy. In Thailand, Singtong et al. (2021) [36] found that targeted programs improved secondary school students’ understanding of food labels, contributing to healthier eating behaviors. Similarly, in Indonesia, food literacy initiatives enhanced food label comprehension and promoted healthier dietary choices among high school students (Dwijayanti et al., 2021; Safitri & Rahayu, 2018) [37,38]. However, these studies did not specifically categorize literacy levels into low, moderate, or high. Despite this, their findings suggest that food label literacy interventions can effectively promote healthier food choices. These insights highlight the potential for similar initiatives in Malaysia to improve adolescents’ food label literacy and dietary behaviors.

The Games-Howell post hoc test revealed significant regional differences in Comprehension & Interpreting Food Labels, Skills Using Food Labels, and Attitudes Toward Food Labels, indicating disparities in food label literacy across regions. For comprehension and interpreting food labels, participants in Central had significantly lower scores than those in Southern, Northern, and East, suggesting possible differences in education, awareness, or consumer behavior. Additionally, Southern scored lower than East and East Malaysia, highlighting potential gaps in food label comprehension across regions. For skills using food labels, Central performed worse than Southern, East, and East Malaysia, while Northern and East outperformed Central. Although consumers may be aware of the benefits of nutrition labels, they often do not refer to them when purchasing food products. This behavior contributes to unhealthy food choices and an increased risk of health problems [11]. This could explain why participants from Central performed worse in skills using food labels compared to other regions, as they may lack the habit of actively using nutrition labels when making food choices. This may explain why Central performed worse, as they may not habitually use nutrition labels when choosing food. Also, a review found that consumers with prior nutrition knowledge use food labels more effectively [39], suggesting that better-performing regions like Northern and East may have a population with higher nutrition knowledge, allowing them to interpret and utilize food labels more effectively in their decision-making process. As for attitudes toward food labels, Central had significantly lower scores than Northern, East, and East Malaysia, while Southern scored lower than Northern and East. This suggests that attitudes toward food labels vary by region, potentially influenced by media exposure, public health policies, or consumer awareness. Overall, this indicates a need for targeted interventions in lower-performing regions, particularly Central and Southern, to enhance food label literacy and encourage informed dietary choices.

Apart from that, the findings of this study reveal that the mean scores for all three domains tend to increase with age. The lower scores suggest a lesser understanding of food labels, which is potentially influenced by the fact that most food purchases are made by their parents. These results align with the research by Zhang et al. (2017) [40] and Mengi et al. (2023) [41], who showed age-related variations in literacy levels. This trend may be attributed to a growing interest in nutrition and health, coupled with extensive exposure [42]. Additionally, a study suggests that nutrition literacy tends to improve with age [34]. As anticipated, age emerged as a significant factor in food label literacy, with older adolescents demonstrating higher literacy levels. While the expected increase in food label literacy with age was observed, it was unexpected that socioeconomic status was significantly associated with understanding, but not with skills or attitudes. This finding suggests that factors beyond household income such as school-based education or media exposure may play a more influential role in shaping label-related behaviors, as recent studies have shown that such interventions can significantly improve adolescents’ food label literacy, often independently of socioeconomic status [43,44]. Additionally, the lack of significant sex differences in skills and attitudes was surprising, especially given that previous studies often report greater engagement among females. Supporting this, Rafiya et al. (2024) [45] found that although adolescent girls had varying levels of knowledge about food labels, this did not significantly influence their attitudes or practices. These findings suggest that increasing knowledge alone may not be sufficient to change behavior, highlighting the need for targeted intervention programs to improve label literacy. No significant difference was observed in BAZ concerning the level of nutrition label literacy. This finding is aligned with a study reporting that reading food labels is inversely associated with BMI [46]. In addition, another study reported that food label reading was not significantly influenced by BMI [31]. The mean score readings for the obese category in the attitude towards food labels domain are high, possibly because they want to avoid foods or nutrients that may pose health risks, as informed by [21].

## Conclusion

In conclusion, although the prevalence of food label reading among adolescents in Malaysia is high at 89.7%, the level of food label literacy among adolescents aged 13–16 in schools in Malaysia is at a moderate level. When comparing the level of food label literacy among adolescents across five zones in Malaysia with sociodemographic factors, a significant relationship (p < 0.05) was found between age and the level of food label literacy, indicating higher literacy values in older adolescents. Likewise, skills in using food labels showed a significant difference based on the father’s educational level. In an “obesogenic” environment that disrupts the balance between calorie intake and energy expenditure [47], many Malaysian children from various sociodemographic backgrounds do not meet dietary recommendations [48]. While our study emphasizes the role of food label literacy in fostering healthier food choices, it does not establish a direct causal link between literacy and obesity risk. However, considering that obesogenic environments contribute to poor eating habits, enhancing food label literacy could be an important strategy to mitigate obesity risk.

Thus, an intervention study to educate adolescents on nutrition labels is crucial to promote healthier food choices and address these dietary deficiencies. A crucial gap in health education can be addressed by fostering proper comprehension, skills, and attitudes toward food labels. This can be achieved through initiatives, such as school-based educational campaigns or social media campaigns that actively engage adolescents, helping them understand the importance of reading food labels and interpreting them to facilitate informed dietary practices. School-based campaigns should integrate food label education into the curriculum through interactive hands-on activities like supermarket tours, and peer-led initiatives. Parental and teacher involvement, along with digital tools such as apps and social media challenges, can further enhance engagement, fostering a sustainable approach to improving food label literacy among adolescents. Future research should further explore this potential relationship.
